# Impacts of sulfate polysaccharide JCS1S2 on retinal neovascularization in oxygen-induced retinopathy rats

**DOI:** 10.3389/fphar.2025.1499420

**Published:** 2025-04-09

**Authors:** Yang Bai, Rui Wang, Can Jin, Lili Wang, Yun Tang, Han Wang, Kan Ding, Shanjun Cai

**Affiliations:** ^1^ Department of Ophthalmology, Special Key Laboratory of Ocular Diseases of Guizhou Province, Affiliated Hospital of Zunyi Medical University, Guizhou Eye Hospital, Guizhou Provincial Branch of National Eye Disease Clinical Research Center, Zunyi Medical University, Zunyi, Guizhou Province, China; ^2^ Zhongshan Institute for Drug Discovery, Shanghai Institute of Materia Medica, Chinese Academy of Science, Zhongshan, China; ^3^ Glycochemistry and Glycobiology Lab, CAS Key Laboratory of Receptor Research, State Key Laboratory of Drug Research, Shanghai Institute of Materia Medica, Chinese Academy of Sciences, University of Chinese Academy of Sciences, Beijing, China

**Keywords:** *Dendrobium nobile Lindl*, polysaccharide sulfate JCS1S2, retina, oxygen-induced retinopathy, vascular endothelial growth factor

## Abstract

Retinal neovascularization, a pathological form of angiogenesis, is a leading cause of blindness. This study investigated the effects of Dendrobium sulfate polysaccharide JCS1S2, derived from *Dendrobium chrysogenum*, on oxygen-induced retinopathy (OIR) in rat models and Müller cells of the rat retina. To this end, we established an OIR rat model and divided the rats into three primary groups, namely, Group I (control), Group II (OIR), and Group III (OIR + JCS1S2). Group III was further subdivided into three subgroups treated with different concentrations of JCS1S2 (10 μg/μL, 20 μg/μL, and 40 μg/μL). After finding the optimal concentration of JCS1S2 by ADP and HE, PCR, Western blot and transcriptome sequencing were used to analyze the role of JCS1S2 in Müller cells of OIR rats and rat retinas. ADP and hematoxylin and eosin (HE) staining revealed that JCS1S2 dose-dependently inhibited retinal neovascularization. Quantitative polymerase chain reaction (qPCR) and Western blot analyses showed significant downregulation of vascular endothelial growth factor A (VEGF-A), VEGF-B, VEGF-D, VEGF receptor 1 (VEGF-R1), and VEGF receptor 2 (VEGF-R2) following JCS1S2 treatment. Transcriptome analysis suggested that JCS1S2 may suppress the activation of the Toll-like receptor (TLR) signaling pathway, regulate the expression of genes associated with endothelial activation and angiogenesis, and participate in the inflammatory and metabolic pathways of the retina. Western blotting data indicate that JCS1S2 can markedly reduce abnormal retinal angiogenesis and Müller cell activation in OIR rats through the TLR4/p-NF-κB/VEGF pathway, JCS1S2 may have the potential as a therapeutic agent for retinal neovascularization.

## 1 Introduction

Retinal neovascularization is a predominant clinical feature of retinopathy of prematurity (ROP) (Hyland et al., 2013) and a significant contributor to blindness across various pathologies, including diabetic retinopathy (DR) and age-related macular degeneration (AMD) (Evans et al., 2020; Sun et al., 2019). These aberrant blood vessels can infiltrate the choroidal vessels, the retinal photoreceptor layer, and other normal fundus tissues, thereby impairing visual function. In cases of ROP, the uncontrolled growth of retinal neovascularization can lead to hemorrhage and retinal traction, causing holes and detachment, which may potentially result in permanent blindness in severe cases (Sapieha et al., 2010). The management of retinal neovascularization is critical for the treatment of ROP (Leng et al., 2018). Vascular endothelial growth factor (VEGF) plays a central role in angiogenesis and the progression of ROP, contributing to enhanced vascular permeability, neovascularization, and the proliferation of pathological endothelial cells (Movsas and Muthusamy, 2020; Robbins et al., 1998). The VEGF family consists of six isoforms, namely, VEGF-A, VEGF-B, VEGF-C, VEGF-D, VEGF-E, and placental growth factor (PIGF). VEGF receptors (VEGFRs) are cell surface receptors for VEGF and are essential in regulating angiogenesis, vascular development, vascular permeability, and embryonic hematopoiesis. VEGF ligands bind to three main transmembrane endothelial receptors, namely, VEGF receptor 1 (VEGF-R1), VEGF receptor 2 (VEGF-R2), and VEGF receptor 3 (VEGF-R3) (Berg et al., 2015; Melincovici et al., 2018). The binding of VEGF to VEGFRs initiates the formation of receptor dimers and activates downstream signaling pathways, leading to a rapid increase in intracellular calcium ion concentration and the production of inositol triphosphate. This cascade modulates the growth of vascular endothelial cells, promotes their proliferation and migration, alters the extracellular matrix, and induces retinal neovascularization (Jia et al., 2004).

VEGF-A is most prominently associated with ophthalmological conditions and is acknowledged as the primary mediator in angiogenesis and the progression of disorders such as DR and AMD. Its principal functions encompass enhancing vascular permeability, fostering neovascularization, and facilitating the proliferation of pathological endothelial cells (Melincovici et al., 2018; Khanani et al., 2020). In conjunction with VEGF-A, other growth factors, including VEGF-B and PIGF, are also involved in these angiogenesis-related pathological processes (Mesquita et al., 2018; Lennikov et al., 2020). Research has underscored the critical role of the VEGF receptor VEGF-R2 in the pathogenesis of various angiogenic processes, including ROP, by modulating the proliferation of retinal vascular endothelial cells through diverse signaling pathways (Simmons et al., 2018). Intravitreal administration of anti-VEGF agents effectively prevents the binding of VEGF to its receptors, thereby abrogating retinal neovascularization and diminishing the risk of fundus hemorrhage (Sankar et al., 2016). However, clinical outcomes for many patients do not correspond to the degrees of benefit observed in clinical trials, partly attributable to a lower frequency of anti-VEGF injections. The short half-life of currently available anti-VEGF medications and the requirement for frequent intraocular injections, particularly for conditions like age-related macular degeneration, present considerable challenges. These challenges are compounded by the potential risk of endophthalmitis, underscoring the urgent need for the discovery and development of novel, long-acting VEGF inhibitors.

The Dendrobium genus, a distinguished member of the Orchidaceae family, is abundant in bioactive constituents, including polysaccharides, flavonoids, alkaloids, dendrobium phenols, amino acids, phenanthrenes, and benzyl derivatives. These compounds possess a broad spectrum of pharmacological activities, notably antioxidant activity. Dendrobium has been documented in the Chinese Pharmacopoeia since approximately 200 A.D., where it is recognized for its astringent, analgesic, tonic, and anti-inflammatory properties. Its medicinal history extends back to the Qin and Han Dynasties, and it is celebrated in the “Shennong’s Herbal Classic” as the “preeminent among the nine immortals” of esteemed Chinese herbal medicines (Yuan et al., 2019; Lam et al., 2015). Dendrobium is renowned for its benefits in gastrointestinal health, renal nourishment, and ocular brightening (Wang et al., 2010). Recent investigations have revealed that Dendrobium polysaccharides exhibit anti-diabetic, anti-angiogenic, anti-cancer, anti-oxidative, anti-inflammatory, and immunomodulatory effects (Liang et al., 2021; Gu et al., 2021; Yang et al., 2021; Zhao et al., 2017). Our research has extracted and purified six neutral polysaccharides (JCS0, JCS1, JCN1-1, 1AE1, 1AE2, and B1) and one acidic polysaccharide (JCS2S1) from the dried stem of *Dendrobium nobile Lindl.* Furthermore, we synthesized JCS1S2, a sulfated derivative of JCS1, which shows promising efficacy in inhibiting vessel proliferation. Bioactivity assessments have demonstrated that JCS1S2 can suppress the lumen formation of human microvascular endothelial cells by reducing VEGF expression, thereby exhibiting anti-angiogenic properties (Wang et al., 2019). We propose that the binding of JCS1S2 to the vitreous and other ocular structures may serve as a strategy to impede the retention of VEGF within the eye, potentially alleviating the burden of intravitreal injections. Using animal models and pathological studies, we found that JCS1S2 can inhibit retinal neovascularization in OIR rats. Through transcriptome sequencing, we explored the gene expression alterations during OIR progression and treatment, revealing the complex interplay between angiogenesis, inflammation, neurotrophic factors, and cellular metabolism. This study aims to identify the pivotal targets of JCS1S2 in the treatment of OIR. Using an OIR rat model and *in vitro* cultured retinal Müller cells under simulated hypoxia, we assessed the pathway effects, elucidating the specific impact of JCS1S2. This research establishes a theoretical framework for the early treatment and prognostic investigation of ROP and offers innovative perspectives for the clinical management of ocular disorders.

## 2 Materials and methods

### 2.1 Preparation of polysaccharides and animals

Dried stems of *Dendrobium nobile* were procured from *Dendrobium nobile* Industrial Development Co., Ltd., situated in Chishui, Guizhou, China. JCS1S2 was prepared in accordance with the methodology delineated in the prior literature (Wang et al., 2019; Jin et al., 2017). The molecular framework of JCS1S2 is composed of 1,4-linked β-Manp and 1,4-linked α-Glcp residues, with sulfation occurring at the O-6 positions of both β-Manp and α-Glcp. The degree of sulfation is 1.74, and the molecular weight is 56.2 kDa, as documented by Wang et al. (2019). The Experimental Animal Center at Zunyi Medical University provided all the suckling Sprague-Dawley (SD) rats used in the trial, and they were of grade SPF according to the national standard for the use of medicinal animals and in compliance with the ARVO statement for the use of animals in ophthalmic and vision research. This study was approved by the university’s ethics committee (Ethics No.: KLLY (A)-2021-107).

### 2.2 Oxygen-induced retinopathy rat model and intravitreal injection of JCS1S2

**FIGURE 1:**
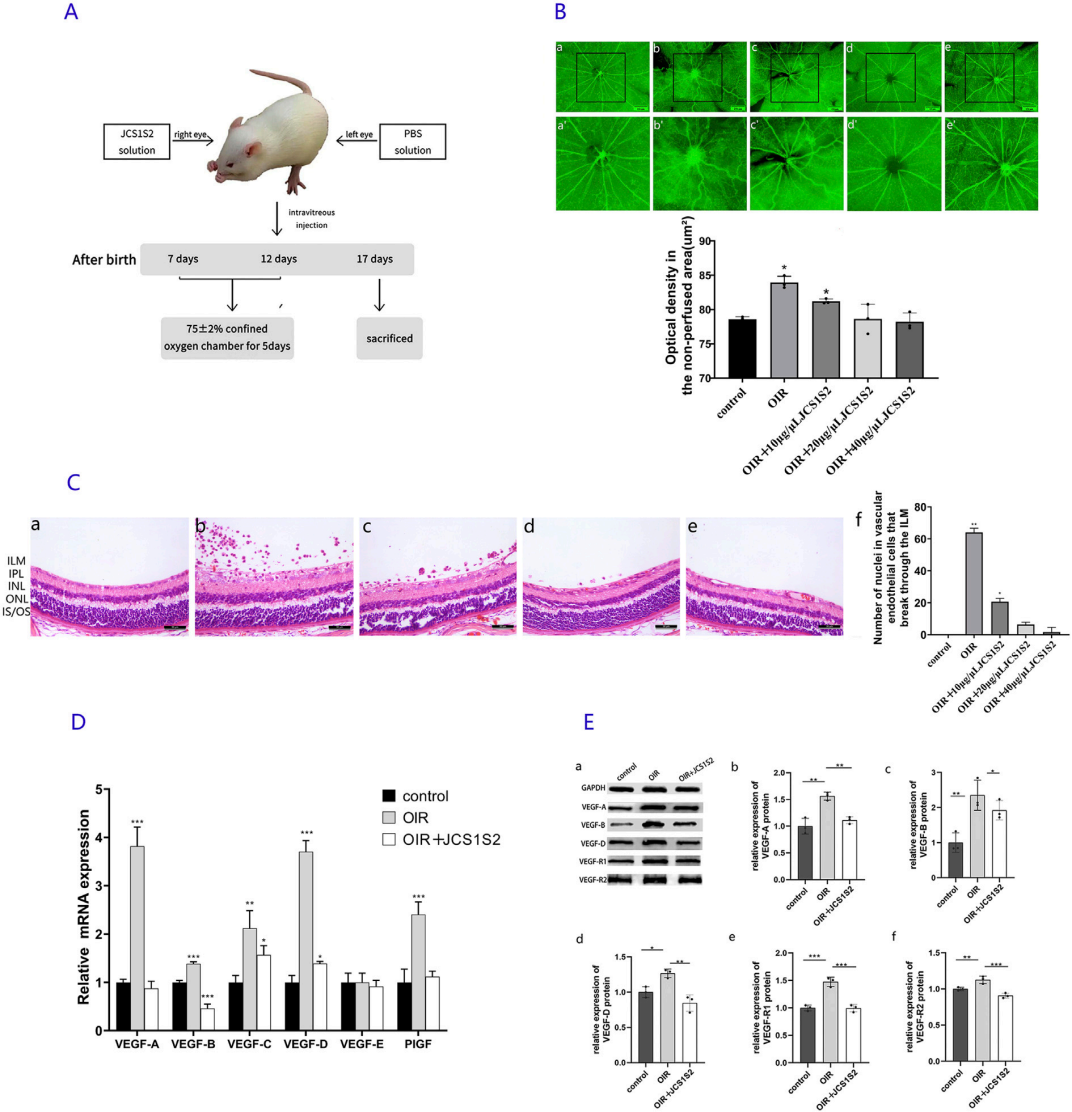
**(A)** Experimental model diagram. **(B)** ADP staining image. **(a)** In the control group, retinal vessels are arranged radially, centered on the optic disc, and distributed uniformly and symmetrically in the quadrants. The vessels appear normal, without any signs of disorder or distortion. **(b)** In the OIR group, an area of non-perfusion is evident, with large vessel branches becoming tortuous, localized vessel dilation, and small branches clustering into knots. Extensive neovascularization is visible throughout the retina. **(c–e)** JCS1S2 partially mitigates these lesions, with **(c)** OIR + 10 μg/μL JCS1S2, **(d)** OIR + 20 μg/μL, and **(e)** OIR + 40 μg/μL. **(a’–e’)** Areas of avascular perfusion selected with **(a–e)** as the central areas (scale: 250 μm; *P < 0.05 vs. control group). **(C)** HE staining of retinal tissue. **(a)** Control group, **(b)** OIR group, **(c)** OIR + 10 μg/μL JCS1S2, **(d)** OIR + 20 μg/μL JCS1S2, and **(e)** OIR + 40 μg/μL JCS1S2. ILM, inner limiting membrane; IPL, inner plexiform layer; INL, inner nuclear layer; ONL, outer nuclear layer; IS/OS: cone rod layer (scale: 30 μm; *P < 0.05 vs. control group and **P < 0.01 vs. control group). **(D)** mRNA expression levels of VEGF-A, VEGF-B, VEGF-C, VEGF-D, VEGF-E, and PIGF in the control, OIR, and OIR + 20 μg/μL JCS1S2 groups (*P < 0.05 vs. control group, **P < 0.01 vs. control group, and ***P < 0.001 vs. control group). **(E)** Protein expression levels of VEGF-A, VEGF-B, VEGF-D, VEGF-R1, and VEGF-R2 in the control, OIR, and OIR + 20 μg/μL JCS1S2 groups (*P < 0.05 vs. control group, **P < 0.01 vs. control group, and ***P < 0.001 vs. control group).

In this study, 138 suckling SD rats were used. Initially, 30 newborn SD rats were randomly allocated into three groups (n = 10 per group), namely, control, OIR, and OIR + JCS1S2. The JCS1S2 group received intravitreal injections of JCS1S2 at concentrations of 10 μg/μL, 20 μg/μL, and 40 μg/μL, according to the relevant modeling method (Said et al., 2017; Yoon et al., 2009). The control group was maintained in ambient air until postnatal day 17 (P17). The OIR model was induced by placing pups in a hyperoxic chamber (75% ± 2% oxygen) from P7 to P12, followed by a return to room air until P17. On P12, the OIR + JCS1S2 group received JCS1S2 injections, while the control and OIR groups received PBS injections. Following the determination of the optimal JCS1S2 concentration based on histopathology, an additional 108 rats were divided into control, OIR, and OIR + JCS1S2 (optimal concentration) groups (n = 36 per group) for further experiments. The birth weights of rats in the control, OIR, and OIR + JCS1S2 groups were 6.03 ± 0.53 g, 5.96 ± 0.58 g, and 6 ± 0.51 g, respectively. The body weights on day 7 were 15.99 ± 0.8 g, 15.91 ± 0.93 g, and 15.94 ± 0.89 g, respectively. The body weights on day 12 were 34.14 ± 1.66 g, 34.37 ± 1.75 g, and 34.32 ± 1.45 g, respectively. The body weights on day 17 were 50.55 ± 2.5 g, 50.68 ± 2.29g, and 50.82 ± 2.22 g, respectively. The experimental timeline is depicted in Figure 1A.

On postnatal day 12, mydriatic drugs were administered to the right eyes of the suckling rats. A single operator performed all injections using a Hamilton 700 microsyringe (10 μL, Reno, United States) and a 33-gauge needle, carefully avoiding the lens by injecting obliquely behind the keratoscleral margin. A 4 μL solution was injected into the vitreous cavity, followed by the application of a small amount of erythromycin eye ointment to the conjunctiva. On day 17, rats were anesthetized intraperitoneally and euthanized by cervical dislocation. Immediately thereafter, the eyeballs were excised and immersed in precooled (4°C) normal saline. Retinal tissue was then dissected on an ice block. The cornea and sclera were incised at the limbus, and the cornea was circumferentially scored and removed. The lens and vitreous were then extruded by gentle pressure and carefully excised with forceps. The retina was separated from the sclera using ophthalmic forceps, ensuring careful handling to avoid damage, and finally transferred into a frozen tube for further processing.

### 2.3 Cell culture

They were allocated into four groups. Group A (normal) was cultured under standard conditions (95% air and 5% CO_2_) for 48 h. Groups B, C, and D were subjected to relative hypoxia, with different durations of hyperoxia followed by normoxia: Group B (hyperoxia 12 h + normoxia 12 h), Group C (hyperoxia 24 h + normoxia 24 h), and Group D (hyperoxia 48 h + normoxia 48 h). One day before the model construction and the start of the experiment, logarithmically growing Müller cells were seeded onto six-well plates, and their confluence was confirmed under an optical microscope to ensure healthy growth. Group A was incubated at 37°C, 5% CO_2_, and saturated humidity for 48 h. The relative hypoxia groups (B, C, and D) were initially exposed to a hyperoxic environment (85% O_2_ and 5% CO_2_) for 12, 24, or 48 h, respectively, followed by normoxic conditions for the same durations before terminating the culture. Western blotting was used to detect VEGF expression, aiming to identify the optimal time for the relative hypoxia cell model and verify the expression of related proteins in this model.

### 2.4 Indexes and methods of analysis

Retinal ADP enzyme activity and hematoxylin and eosin (HE) staining were assessed in five experimental groups, namely, control, OIR, OIR + 10 μg/μL JCS1S2, OIR + 20 μg/μL JCS1S2, and OIR + 40 μg/μL + JCS1S2 groups. Following the determination of the optimal JCS1S2 concentration, the groups were reorganized into three groups, namely, control, OIR, and OIR + JCS1S2 (optimal concentration) groups. Subsequently, real-time quantitative reverse transcription polymerase chain reaction (RT-qPCR), Western blotting, RNA sequencing (RNA-seq), and bioinformatics analysis were conducted to evaluate the effects of JCS1S2 treatment.

#### 2.4.1 Analysis of retinal ADP enzyme

On P17, the eyeballs were excised and fixed in 4% paraformaldehyde for 24 h. The retina and choroid were meticulously dissected under an operating microscope. Retinal ADP enzyme activity was measured as described previously (Lutty and McLeod, 1992). The tissues were subjected to sequential rinsing, consisting of five 15-min washes with Tris-maleic acid buffer (0.05 mol/L, pH 7.2), followed by incubation in the ADP reaction solution at 37°C in a constant-temperature incubator. After a 10% sulfuric acid chromogenic reaction, the tissues were again rinsed with Tris-maleic acid buffer. The retinal vessels were visualized by sealing the film with glycerol gelatin, and their morphology and distribution were examined under a microscope.

#### 2.4.2 Analysis of HE staining of the retina

On P17, the eyeballs were harvested and immersed in 4% paraformaldehyde at 4°C overnight for fixation. Subsequently, routine procedures were followed for dehydration, clearing, wax infiltration, and paraffin embedding, resulting in continuous sections. HE staining was conducted after conventional dewaxing of the sections. The number of vascular endothelial cell nuclei penetrating the internal limiting membrane (ILM) was quantified in each section. A single observer performed the counting, randomly selecting three fields of view per section for analysis.

#### 2.4.3 Quantitative RT-PCR

Total RNA was isolated using a standard TRIzol-based method (Invitrogen, United States). Complementary DNA (cDNA) synthesis was carried out using the PrimeScript™ RT Reagent Kit (TaKaRa, Japan), following the manufacturer’s instructions. Real-time quantitative polymerase chain reaction (qPCR) was then performed using the SYBR Green dye on a CFX 96 real-time PCR detection system (Bio-Rad Laboratories, Inc., United States). Glyceraldehyde-3-phosphate dehydrogenase (GAPDH) was used as an internal control, and the relative expression levels of target genes were calculated using the 2^−ΔΔCT^ method. The primer sequences are detailed in Table 1.

**TABLE 1:** Primer design table.

Gene	Forward primer (5′-3′)	Reverse primer (5′-3′)
VEGA-A	GTT​GTG​GAA​GGT​CAG​TTC​AGG​ATG​G	AGT​GGT​AGG​GCA​GAC​AGA​AAG​GG
VEGF-B	GTG​AAG​CCA​GAC​AGG​TGA​GTT​CC	TCA​GGA​GAG​AGG​AGA​AAG​CAG​AAG​G
VEGF-C	CCA​ATT​CTC​CTG​CCT​CAA​CCT​TCC	GTG​ATG​CGA​TGA​CTT​GCT​AAC​TTG​C
VEGF-D	CTG​ACT​GCA​GCA​ACG​GCA​AA	TCA​GCT​TTC​GTG​GTT​CGG​GT
VEGF-E	TGT​GCC​TTC​TTC​CTC​CTC​TTC​CTC	GCT​CAG​TCA​TGC​CTC​CCT​TGT​TC
PIGF	ACT​GCT​GGG​CTC​TTC​TTT​ACC​TTT​G	TCC​TCG​CTA​CAC​CTG​CTC​TTC​C
GAPDH	GAC​ATG​CCG​CCT​GGA​GAA​AC	AGC​CCA​GGA​TGC​CCT​TTA​GT

### 2.5 RNA-seq and bioinformatics analysis

On P17, nine rats from each group were randomly selected and euthanized under anesthesia. The eyeballs were promptly excised and immersed in liquid nitrogen, followed by storage at −80°C. The rat eyeball specimens were subjected to gene chip analysis using the services of Jiangsu Anshengda Biotechnology Company. High-throughput sequencing was conducted using the Illumina NovaSeq 6000 platform, adhering to the manufacturer’s protocols. Differentially expressed genes (DEGs) were identified using DEGseq, applying the negative binomial distribution model for detection and estimating dispersion and logarithmic fold change (Love et al., 2014). Differential gene screening was based on fold change values and P-values, with a threshold of | log_2_Foldchange | ≥ 1 and a significance level of P < 0.05. The selected DEGs underwent gene ontology (GO) annotation and Kyoto Encyclopedia of Genes and Genomes (KEGG) pathway analysis using the hypergeometric test. Triplicate analyses were performed for validation.

### 2.6 Western blotting verifies the expression levels of related proteins in the OIR and relative hypoxia cell models

Retinal tissue was extracted using a highly efficient RIPA buffer, followed by ultrasonic fragmentation on ice to ensure complete protein degradation. Protein concentration was determined using the BCA protein assay method. Equal amounts of protein were separated on a 10% sodium dodecyl sulfate–polyacrylamide gel and transferred to a nitrocellulose membrane (Bio-Rad, Shanghai, China). The membrane was blocked with 5% Tris-buffered saline containing Tween-20 (TBST) and bovine serum albumin at room temperature. Specific primary antibodies against VEGF-A (1: 1000, Cat No. 19003-1-AP, Proteintech), VEGF-B (1: 750, AF7019, Affinity), VEGF-D (1:1000, Cat No. 26915-1-AP, Proteintech), VEGF-R1 (1:1000, AF6204, Affinity), VEGF-R2 (1:1000, AF6281, Affinity), and GAPDH (1: 10,000, Cat No. 60004-1-Ig, Proteintech) were used for incubation. After overnight incubation at 4°C, the membrane was washed three times with TBST and then incubated with horseradish peroxidase (HRP)-conjugated secondary antibody. For pathway studies, retinal tissue and cell samples from rats were treated similarly, adding appropriate primary antibodies (e.g., VEGF, GAPDH, TLR4, and p-NF-κB) and incubating overnight at 4°C. The samples were washed with TBST and incubated with HRP-conjugated secondary antibody overnight. The optical density of each band was measured using a gel imaging system (Bio-Rad Laboratories Inc., United States), and protein band quantification was performed using ImageJ software.

### 2.7 Data analysis

In this study, ImageJ and Image-Pro Plus software programs were used for data analysis, while IBM SPSS Statistics 29.0 (IBM Corp., Armonk, NY, United States; version 29.0) was used for statistical analysis. GraphPad Prism 9 (GraphPad, United States) was used for graphical representation. The Kolmogorov–Smirnov (K-S) test was used to assess normal distribution, and data adhering to normal distribution are presented as the mean ± standard deviation (*x̄* ± SD). For parameters satisfying normal distribution, a one-way analysis of variance (ANOVA) was conducted to compare multiple groups, assuming homogeneity of variances. Post hoc comparisons were performed using the least significant difference (LSD) method for pairs of groups. In cases where variance heterogeneity was present, Tamhane’s T2 method was used for pairwise comparisons. Statistical significance was set at P < 0.05.

## 3 Results

### 3.1 JCS1S2 inhibits retinal angiogenesis in OIR rats

#### 3.1.1 Retinal ADP enzyme

Under normoxic conditions, examination of ADP-stained retinal sections revealed well-branched retinal vessels with a radially uniform distribution of large vessels originating from the optic disc. The vessel diameters were consistent, and there were no perfusion deficits or neovascularization observed in the retina. In contrast, the retinal vessels of P17 rats in the OIR group appeared tortuous and dilated, with a significant area of non-perfused retina observed at the center. Compared to the OIR group, the OIR + JCS1S2 group exhibited a marked reduction in vessel tortuosity and irregular dilation and a significant decrease in non-perfused areas (Figure 1B). The optical density of the non-perfused retinal area was quantified using Image-Pro Plus. ANOVA was conducted to compare the differences among the five groups (F = 18.31; P < 0.05). The retinal vascular perfusion areas in the OIR group and the OIR + 10 μg/μL JCS1S2 group were increased compared to that in the control group (P < 0.05). No significant differences were found between the OIR + 20 μg/μL and OIR + 40 μg/μL JCS1S2 groups and the control group (*P*_20 μg/μL_ = 0.977 and *P*_40 μg/μL_ = 0.720).

#### 3.1.2 HE staining of the retina

Under light microscopy, HE staining of the control group revealed no vascular endothelial nuclei extending through the internal limiting membrane into the vitreous body (Figure 1C). In contrast, the OIR group exhibited a significant number of vascular endothelial nuclei that had penetrated the retinal internal limiting membrane and grown into the vitreous body, either individually or in clusters. Following JCS1S2 injection, there was a marked reduction in the number of vascular endothelial nuclei extending into the vitreous cavity, with a dose-dependent improvement in inhibition. Statistical analysis using Tamhane’s T2 method showed that the number of vascular endothelial cell nuclei breaking through the vitreous cavity in the OIR and OIR + 10 μg/μL JCS1S2 groups was significantly higher than that in the control group (P < 0.05). No significant differences were observed in the number of breakthroughs between the OIR + 20 μg/μL JCS1S2 and OIR + 40 μg/μL JCS1S2 groups compared to that in the control group (*P*_20 μg/μL_ = 0.173 and *P*_40 μg/μL_ = 0.996).

Based on the results from ADP enzyme retinal smears and HE staining (Figures 1B, C), there was no statistical difference between the OIR + 20 μg/μL and OIR + 40 μg/μL JCAS1S2 groups and the control group. No side effects were observed in either concentration group (no mortality, vitreous hemorrhage, or corneal or lens damage in rats). Consequently, this study selected 20 μg/μL as the concentration for further investigation of other indices, while the 40 μg/μL group will continue to be explored in future studies.

### 3.2 Expression of VEGF mRNA in the retina

To investigate the role of JCS1S2 in the progression of retinal neovascularization, we conducted real-time RT-PCR measurements. Rats were categorized into three groups, namely, control, OIR, and OIR + JCS1S2 (20 μg/μL). On P17, there were significant differences in VEGF-A mRNA (F = 136.541, *P* < 0.05), VEGF-B mRNA (F = 169.175, *P* < 0.05), VEGF-C mRNA (F = 14.999, *P* < 0.05), VEGF-D mRNA (F = 251.686, *P* < 0.05), and PIGF mRNA (F = 35.006, *P* < 0.05) among the three groups. However, there was no significant difference in VEGF-E mRNA (F = 0.247, *P* > 0.05). ANOVA was conducted to compare multiple groups; the OIR group showed upregulated mRNA expression of VEGF-A, VEGF-B, VEGF-C, VEGF-D, and PIGF in retinal tissue (P < 0.05). Following the intravitreal injection of JCS1S2, the expression of VEGF-B mRNA in the OIR + JCS1S2 group was lower than that in the control group (P < 0.05). The mRNA levels of VEGF-A, VEGF-B, VEGF-C, VEGF-D, and PIGF in other groups were decreased compared with those in the OIR group (*P* < 0.05). By calculating the multiple difference ratio of relative mRNA expression, we found that the OIR group, compared to the JCS1S2 group, exhibited the most significant reduction in VEGF-A, VEGF-B, and VEGF-D levels following the intravitreal injection of JCS1S2. Detailed results for each group are presented in Figure 1D.

### 3.3 Expression of VEGF-related proteins in the retina

To validate the data, Western blotting was performed to detect VEGF-related protein expression in the rat retina (Figure 1E). Proteins were extracted from the retinas of suckling rats on P17 and analyzed for VEGF-A, VEGF-B, VEGF-D, VEGF-R1, and VEGF-R2. ANOVA was conducted to compare the three groups, which revealed significant differences in the expression of VEGF-A (F = 25.215), VEGF-B (F = 12.701), VEGF-D (F = 17.489), VEGF-R1 (F = 48.445), and VEGF-R2 (F = 24.675) proteins (P < 0.05). The OIR group showed a significant upregulation of VEGF-A, VEGF-B, VEGF-D, VEGF-R1, and VEGF-R2 protein expression compared to the control group (P < 0.05). Following the intravitreal injection of JCS1S2, the expression of these proteins was decreased in the OIR + JCS1S2 group compared to that in the OIR group (P < 0.05), as depicted in Figure 1E.

### 3.4 Transcriptome analysis reveals key signaling and mediating genes

#### 3.4.1 Differentially expressed genes

**FIGURE 2:**
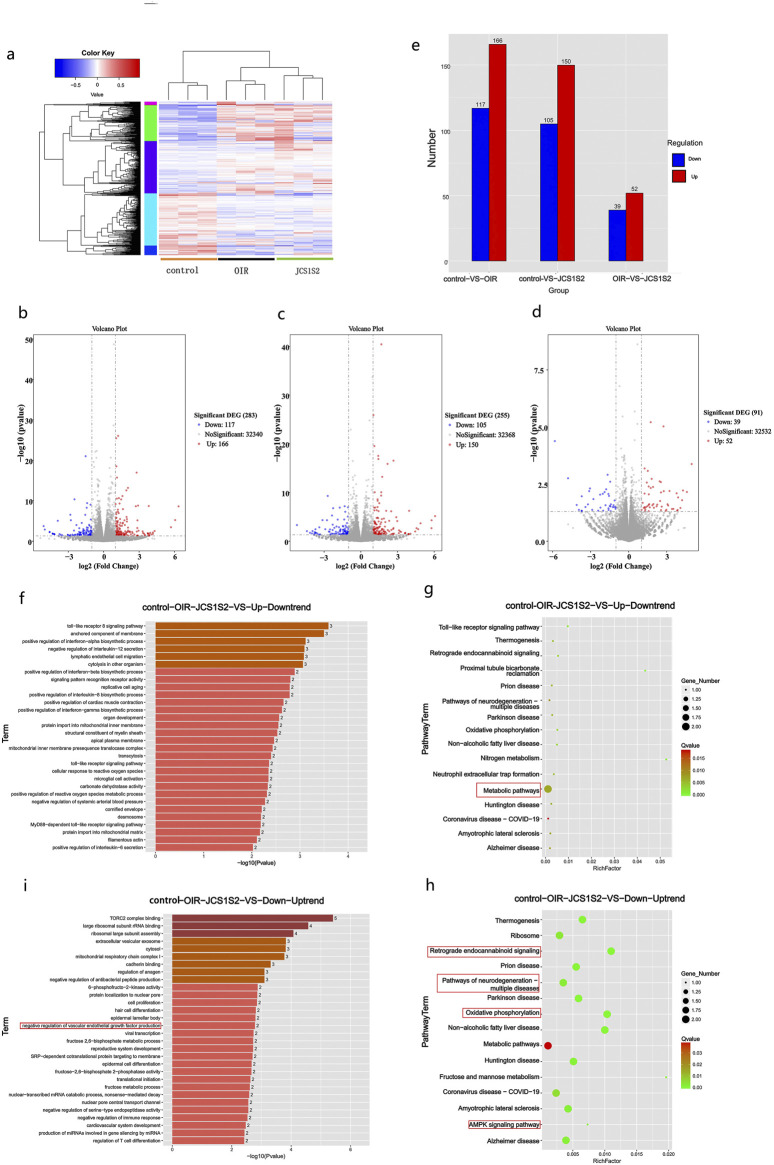
**(a–e)** Heat map note of cluster analysis in each group. The color code at the bottom shows the relative expression level of mRNA in three groups of samples: different color regions indicate distinct clustering groups, with similar gene expression patterns within the same group and distinct patterns between different groups. Red dots denote significantly upregulated genes, blue dots indicate significantly downregulated genes, and black dots represent genes with unchanged expression levels. **(f–i)** GO enrichment analysis: **(f)** genes with an up–down trend, predominantly associated with inflammatory processes. **(i)** Genes with a down–up trend, primarily enriched in TORC2 complex binding. The darkness of the color indicates the level of gene expression, with darker shades representing higher expression. **(g, h)** Bubble diagram of the KEGG pathway analysis. **(g)** Genes with an up–down trend, predominantly involved in metabolic pathways. The KEGG pathway analysis, conducted using Metascape, also reveals the presence of TLR signaling pathway genes, which are consistent with the findings of GO analysis. **(h)** Genes with a down–up trend, associated with pathways related to neurodegeneration, neurodegenerative diseases, oxidative phosphorylation, and the AMPK signaling pathway. Bubble size represents the level of gene expression, with larger bubbles indicating higher expression. Nodes are color-coded from red to green, representing a gradient of −log10 (P-value) significance.

Using the Rat Genome Database, we identified a total of 427 differentially expressed genes across the three groups. The volcano plot, derived from hierarchical clustering analysis, illustrates the distinct mRNA expression patterns among the groups (Figure 2a). The color code at the bottom of Figure 2a indicates the relative expression levels of the three sample groups. Different color regions denote various clustering groupings, with similar gene expression patterns within the same group and divergent patterns between different groups. The significance threshold was set to >2.0 for multiplicative changes, with an adjusted P-value < 0.05. Of the 427 differential genes, 166 were upregulated and 117 were downregulated in the OIR group (Figure 2b), while 150 were upregulated and 105 were downregulated in the JCS1S2 group (Figure 2c). In comparison with the OIR group, 52 genes were upregulated and 39 genes were downregulated in the JCS1S2 group (Figure 2d; Figure 2e). In Figures 2b–d, red dots represent significantly upregulated genes, blue dots indicate significantly downregulated genes, and black dots denote genes with unchanged expression levels. Furthermore, genes exhibiting a fluctuating expression trend across the control, OIR, and JCS1S2 groups were selectively analyzed, revealing a total of nine genes with an up–down trend and nine genes with a down–up trend. The genes with an up–down trend are *Ca4*, *Lgals5*, *Gp2*, *Romo1*, *Ndufa1*, *Tlr8*, *LOC100911027*, *LOC103690128*, and *AABR07050321*.2. The genes with a down–up trend are *LOC100912599*, *RGD1564606*, *AABR07021384.1*, *LOC108348103*, *NEWGENE*_*1359268*, *LOC100911725*, *LOC108348144*, *AABR07001061.1*, and *AABR07018058.1*.

#### 3.4.2 Enrichment analysis of genes and pathways in each group

Gene expression analysis was performed on genes exhibiting up–down trend and down–up trend patterns across the control, OIR, and JCS1S2 groups. This analysis revealed the involvement of these genes in various biological functions and pathways related to JCS1S2’s role in ocular treatment. Genes with an up–down trend were associated with inflammatory processes, including Toll-like receptor (TLR) signaling, tumor necrosis factor-α (TNF-α), interleukin-12 (IL-12), membrane-anchored components, and lymphoendothelial cell migration, among others (Figure 2f). Conversely, genes with a down–up trend were primarily enriched in biological functions such as TORC2 complex binding, ribosome binding, extracellular vesicle exocytosis, and cytoplasmic and troponin binding (Figure 2i). Notably, genes with a down–up trend also showed enrichment in the negative regulation of VEGF production, with AABR07018058.1 being a gene related to VEGF. The results indicated that most DEGs were significantly enriched in cellular components.

KEGG pathway analysis, conducted using Metascape, revealed that TLR signaling pathway genes, which also appeared in the GO analysis, were associated with metabolic pathways in the up–down trend DEGs. In contrast, down–up trend DEGs related to ophthalmology were mainly involved in pathways associated with neurodegeneration, neurodegenerative diseases, oxidative phosphorylation, and the AMPK signaling pathway (Figures 2g, h).

### 3.5 The JCS1S2 pathway was verified by Western blotting

**FIGURE 3:**
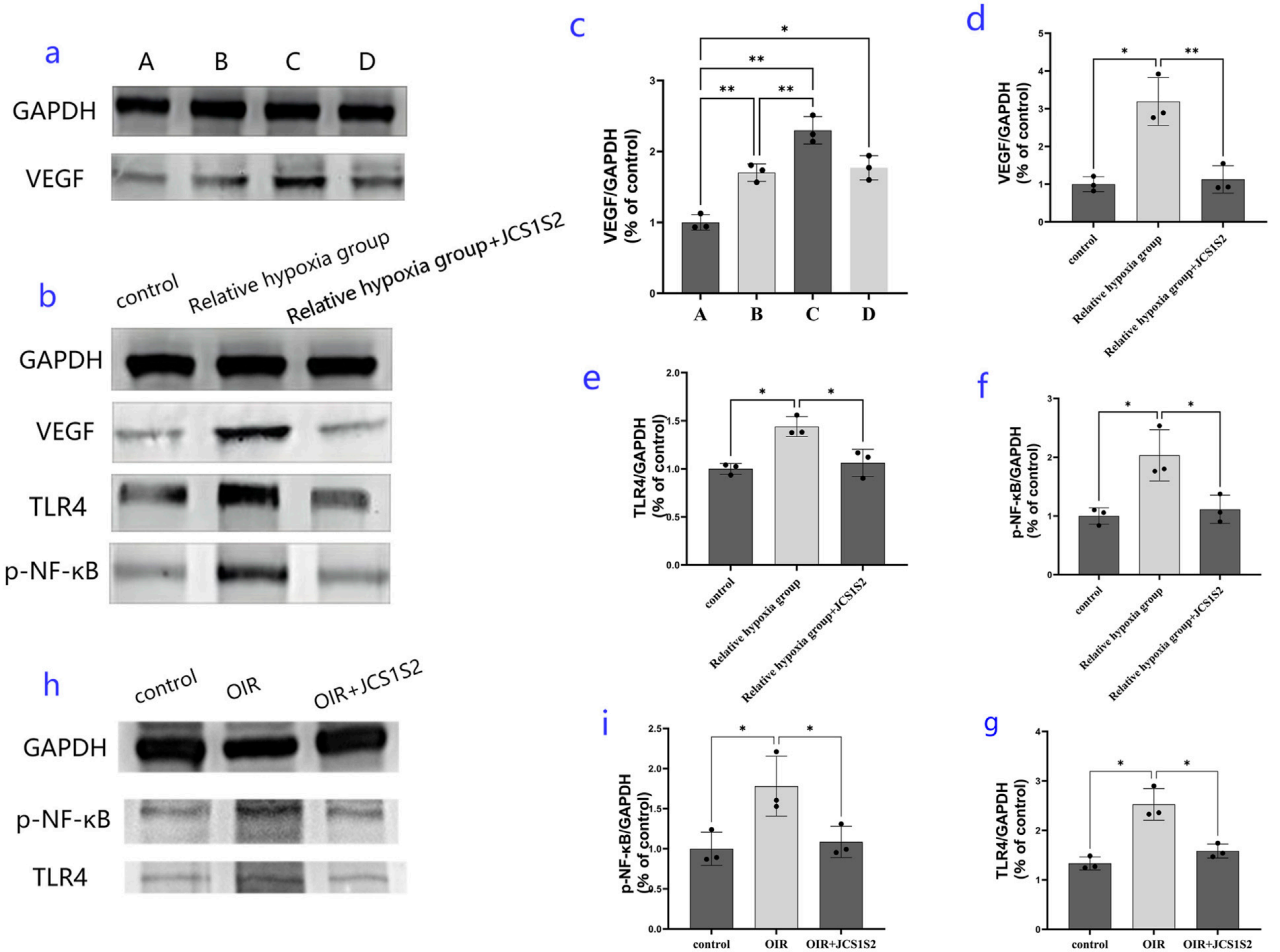
Expression of proteins in each group. **(a, c)** protein expression levels of VEGF in the four groups [normal group and relative hypoxia group (hyperoxia 12 h + normoxic 12 h, hyperoxia 24 h + normoxic 24 h, and hyperoxia 48 h + normoxic 48 h)]; **(b, d, e, f)** protein expression levels in the three groups: expression levels of VEGF, TLR4, and p-NF-κB protein in the normal group, relative hypoxia group (hyperoxia 24 h + normoxic 24 h), and JC1S1S2 group. **(h, i, g)** protein expression results detected in a rat model of OIR (*P < 0.05 and **P < 0.01).

In this study, we utilized the OIR animal model and cultured retinal Müller cells *in vitro* to simulate the relative hypoxic environment of the OIR rat retina by exposing them to hyperoxia followed by normoxia. The expression levels of VEGF in each group of cells were then assessed, as depicted in Figure 3. ANOVA was conducted to compare multiple groups; the expression level of VEGF in the relative hypoxia group was significantly higher than that in the normoxia group. Notably, the expression level of VEGF in groups B, C, and D was significantly elevated compared to that in group A, with group C exhibiting the highest expression, significantly surpassing both groups B and D. These differences were statistically significant (P < 0.05).

Western blotting experiments suggested that the optimal relative hypoxia condition for inducing VEGF expression in Müller cells is 24 h of hyperoxia followed by 24 h of normoxia. Transcriptome sequencing analysis led us to focus on VEGF, TLR4, and p-NF-κB proteins. Western blotting revealed that Müller cells significantly upregulated the expression of VEGF, TLR4, and p-NF-κB proteins in a relatively hypoxic environment. However, administration of the JCS1S2 drug significantly reduced the expression of these proteins, and these changes were statistically significant (P < 0.05).

**FIGURE 4:**
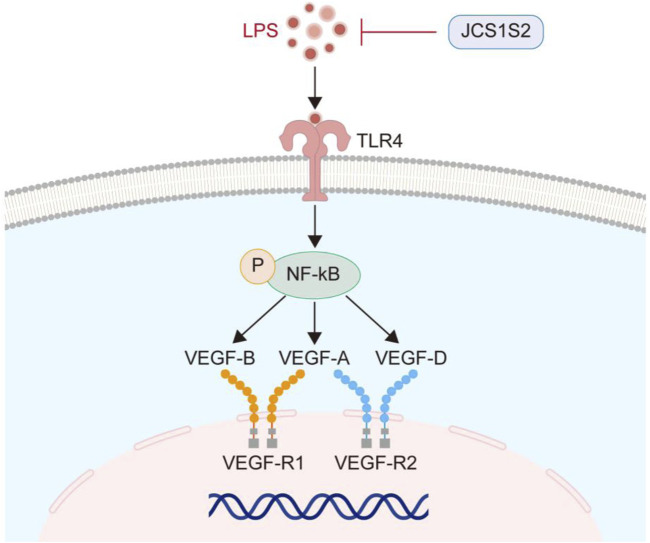
TLR4/p-NF-κB/VEGF pathway.

In the animal models, JCS1S2 administration also led to a significant decrease in the expression of the TLR4 protein and p-NF-κB, with statistically significant differences observed (P < 0.05). To facilitate a clearer understanding, the TLR4/p-NF-κB/VEGF pathway is illustrated in Figure 4.

## 4 Discussion

Retinal neovascularization is a multifaceted pathological process triggered by various stimulatory factors, including ischemia, hypoxia, and inflammation (Rosen, 2002; Kanukollu and Ahmad, 2023; Mones et al., 2021). In retinopathy of prematurity, the immature retinal tissue of preterm infants experiences arrested or delayed vascular growth in avascular areas under hyperoxic conditions. However, upon withdrawal from hyperoxia, the retina in these avascular areas becomes hypoxic, prompting an excessive neovascular response and leading to ROP (Mezu-Ndubuisi et al., 2020; Babaei et al., 2018; Brown and Nwanyanwu, 2023). Hypoxia-ischemia-induced retinal neovascularization is considered to be mainly caused by the increase in VEGF, a potent inducer of angiogenesis and vascular permeability. Hypoxic conditions stimulate various cell types to secrete VEGF (Babaei et al., 2018; Brown and Nwanyanwu, 2023). VEGF is a homologous dimer glycoprotein, which is mainly distributed in retinal pericytes, vascular endothelial cells, and pigment epithelial cells (Wu et al., 2015). The VEGF family comprises six isoforms, namely, VEGF-A, VEGF-B, VEGF-C, VEGF-D, VEGF-E, and PIGF (Berg et al., 2015; Wu et al., 2015). In ophthalmic research, it is found that VEGF-A and VEGF-B are closely related to retinal vessels (Arepalli and Kaiser, 2021; Nishijima et al., 2007; Papadopoulos et al., 2012). VEGF-A is the most effective angiogenic gene, which can affect the important process of angiogenesis and induce endothelial cell proliferation, germination, and lumen formation (Jakeman et al., 1993). The overexpression of VEGF-B promotes the decomposition and destruction of the pathological retina, choroidal neovascularization, and the blood–retinal barrier and increases vascular permeability without causing inflammation, thus participating in the occurrence and development of retinal neovascularization (Zhong et al., 2011). The primary receptors for VEGF ligands are VEGF-R1 and VEGF-R2. VEGF-R1 mediates the migration of endothelial cells and participates in pathological angiogenesis in adulthood (Takahashi and Shibuya, 2005; Neufeld et al., 1999), VEGF-R2 mainly mediates the effects of VEGF-A on mitosis, neovascularization, and vascular permeability (Wu et al., 2017). VEGF-A can bind to both VEGF-R1 and VEGF-R2, whereas VEGF-C and VEGF-D bind to VEGF-R2 and VEGF-R3. PIGF and VEGF-B exclusively bind to VEGF-R1, and VEGF-E interacts with VEGF-R2 (Melincovici et al., 2018; Tamura et al., 2020; Mok et al., 2014). Upon VEGF binding to its receptors, autophosphorylation of the receptors occurs, activating phosphatidylcholine-specific phospholipase and promoting endothelial cell growth and increased vascular permeability (Takahashi and Shibuya, 2005; Wu et al., 2017).

Currently, a variety of medications are available for the treatment of neovascularization in clinical settings (Jhaveri et al., 2022; Holekamp et al., 2022). Anti-VEGF drugs can be divided into two categories, namely, monoclonal antibodies (single target) and receptor fusion proteins (multi-target). The target of monoclonal antibodies is VEGF-A, and the targets of receptor fusion protein are VEGF-A, VEGF-B, and PlGF (Cui et al., 2018). These medications exert their effects by binding to different genes or regions within the VEGF family, potentially eliciting antagonistic actions through various molecular pathways, which may account for the diverse efficacies observed among these drugs.

In the present study, we analyzed the similarities and differences in functions and pathways between JCS1S2 (Wang et al., 2019), a sulfated derivative of *Dendrobium nobile*, and rat retinal VEGF family genes VEGF-A, VEGF-B, VEGF-C, VEGF-D, VEGF-E, PIGF, VEGF-R1, and VEGF-R2. Our objective was to provide a scientific foundation for elucidating the anti-VEGF mechanism of JCS1S2, thereby contributing to the development of novel therapeutic strategies for managing neovascularization-related disorders.

The results of our study indicate that neovascularization is a primary pathological alteration in OIR. Following the intravitreal injection of JCS1S2, we observed a dose-dependent reduction in the number of vascular endothelial nuclei penetrating the internal limiting membrane, a decrease in circuitous dilated vessels, and a reduction in the area of non-vascular perfusion. These findings demonstrate that JCS1S2 effectively inhibits the proliferation of retinopathy-associated vessels. However, this methodology is not without limitations. To enhance the quantification of retinal angiopathy, future studies will employ semi-automatic computer-aided programs to measure vascular curvature. Additionally, the staining process revealed that macrophages, vascular endothelial cells, and other immune cells are intermingled at the lesion site, which may complicate the analysis. In this study, we used the contralateral eye as the control, which could potentially influence the results for the ipsilateral eye. The absence of IB4 staining in our study represents another limitation, affecting the interpretation of the results. These shortcomings will be addressed in subsequent studies.

To elucidate the target and related pathways of JCS1S2’s binding to the VEGF family, we performed real-time quantitative PCR. The results showed that the mRNA expression levels of VEGF-A, VEGF-B, VEGF-C, VEGF-D, and PIGF in the OIR group were higher than those in the control group. The relative expression levels of VEGF-A, VEGF-B, and VEGF-D mRNA in the retina of the JCS1S2 group were most significantly reduced compared to those in the OIR group. Western blotting analysis further revealed that the relative expressions of VEGF-A, VEGF-B, VEGF-D, VEGF-R1, and VEGF-R2 in the retina after JCS1S2 administration were lower than those in the OIR group. These findings suggest that the intravitreal injection of JCS1S2 can inhibit the expression of VEGF-A and VEGF-B/VEGF-R1 and VEGF-A and VEGF-D/VEGF-R2, thereby slowing down the proliferation of pathological blood vessels under OIR conditions.

Through transcriptome sequencing, we found that JCS1S2 has multiple effects on OIR and can inhibit angiogenesis by downregulating the Toll-like receptor pathway. As an important signal transduction pathway participant, TLRs play an important role in immune response, and TLR4 is the earliest discovered and most widely distributed TLR (Zhang et al., 2022). TLR4 was expressed in retinal pigment epithelial cells, photoreceptors, Müller cells, glial cells, and vascular endothelial cells (Wang et al., 2015). Yang et al. (2024) found that inducing TLR4 activation can activate NF-κB, upregulate the level of inflammatory factors, and increase the permeability of retinal blood vessels. One of the core characteristics of ROP disease is the growth of new tubules. VEGF is a key mediator of retinal angiogenesis. The TLR4/NF-κB signaling pathway may directly modulate VEGF expression or influence it indirectly through cytokine production, thereby regulating vascular formation (Zhu et al., 2016). Chen et al. (2019) demonstrated the effect of blocking drugs with TLR4 on OIR diseases, and the results show that it can reduce the non-perfusion area of the retina, inhibit abnormal angiogenesis, and improve blood vessel density. Yukimichi et al. (2017) found that TAK-242, a small cyclohexene derivative, inhibited inflammation through the TLR4/p-NF-κB signaling pathway and reduced astrocyte activation in ganglion cells and inner plexiform layers. Transcriptome sequencing analysis via GO and KEGG revealed that JCS1S2 inhibits pathological neovascularization by downregulating the Toll-like receptor pathway. Therefore, we investigated JCS1S2’s regulatory effects on the Toll-like receptor pathway by assessing VEGF, TLR4, and p-NF-κB protein expression *in vivo* and *in vitro* experiments.

After a comprehensive investigation, it was ascertained that the transition of neonates from the hypoxic milieu of the maternal womb to the oxygen-rich external environment results in a corresponding alteration of the microenvironment surrounding their retinal glial cells. This transition is pivotal in triggering the onset of ROP (Dai et al., 2022). Furthermore, retinal glial cells are integral to the regulation of VEGF expression and provide essential spatial support for the development of retinal vasculature (Zwanzig et al., 2021). As predominant glial cells in the retina, Müller cells are crucial in preserving the homeostasis of the fundus tissue and its functionality (Xu et al., 2023). In the experimental context, JCS1S2 demonstrated efficacy in mitigating the damage inflicted by Müller cells under conditions of relative hypoxia.

In the context of OIR in rats, JCS1S2 effectively suppresses the pathological proliferation of neovascularization. To delineate the influence of the TLR4/p-NF-κB/VEGF pathway activation on vascular endothelial cell dysfunction, an OIR rat model was established, using Müller cells as the representative cell model. The findings indicate that in both the hyperoxic condition of the animal model and the relative hypoxic condition of the cell model, the protein expression levels of TLR4, p-NF-κB, and VEGF are markedly diminished following JCS1S2 treatment. This suggests that JCS1S2 administration may mitigate the hypoxia-induced activation of the TLR4/p-NF-κB/VEGF pathway, and by inhibiting inflammatory mediators and ameliorating oxidative stress, it may downregulate the synthesis and secretion of VEGF, thereby potentially delaying or preventing pathological vascular remodeling.

Transcriptomic sequencing analysis of the OIR model elucidated the multifaceted impact of JCS1S2 on OIR. For analysis, we focused on a single pathway, acknowledging that numerous other pathways warrant investigation. In GO analysis, the control–OIR–JCS1S2 group exhibited both upregulated and downregulated trends for 18 genes, with a notable down–up trend observed for DEGs and VEGF production, specifically associated with the gene *AABR07018058.1*. Our hypothesis posits that JCS1S2 may upregulate *AABR07018058.1* in extracellular vesicles, thereby activating the VEGFR pathway through gene upregulation. Some studies have found that after oxidative damage to the retina, inflammation and cell death increase, and retinal function decreases (Wooff et al., 2020). The function of the down–up trend genes predominantly involves interactions with the TORC2 complex, ribosomes, extracellular vesicle exocytosis, cytoplasm, and troponin binding. Remarkably, JCS1S2 has also been implicated in modulating inflammatory responses, characterized by the downregulation of TLR, TNF-α, IL-12, and others. Additionally, it exhibits potential antioxidant and neuroprotective effects against neurologically relevant factors. KEGG pathway analysis revealed that the intravitreal injection of JCS1S2 induced differential regulation of metabolic pathways compared to the OIR group. Notably, genes associated with oxidative phosphorylation and neurodegeneration pathways were upregulated, while *Ca4* and *Ndufa1* genes were involved in the downregulation of metabolic pathways. The *Ca4* gene is a specific marker of choroidal capillary-restricted endothelial cells (Jiao et al., 2020), and it impacts the treatment of pigmented retinitis and age-related macular degeneration (Chirco et al., 2016; Li et al., 2022). The *Ndufa1* gene and mitochondrial DNA mutations are associated with hereditary optic neuropathy in Leber (Ji et al., 2019; Qi et al., 2004), while its pathophysiology is still poorly understood. These results suggest that JCS1S2 delivery may protect the rat’s vascular endothelial cells by downregulating the *Ca4* and *Ndufa1* genes. However, the main upregulated genes *LOC100912599* and *LOC108348144* are not related to ophthalmology (Daiger et al., 2023), which need further research for clarification.

In conclusion, JCS1S2 demonstrates considerable promise as a therapeutic agent for human retinal neovascularization disorders. In the present investigation, a comparison with the control group, which did not receive JCS1S2 injections, revealed that the signaling pathways involving VEGF-A and VEGF-B/VEGF-R1 and VEGF-A and VEGF-D/VEGF-R2 were effectively inhibited by the intravitreal administration of JCS1S2 in rats. RNA-seq has provided valuable insights into the potential mechanisms underlying the action of JCS1S2. Through both *in vivo* and *in vitro* experiments, it was observed that JCS1S2 can mitigate microvascular pathology and neuronal injury by suppressing the TLR4/NF-κB/VEGF signaling pathway. Currently, research on JCS1S2 merely scratches the surface of its potential applications. Transcriptome sequencing has unveiled factors involved in the pathological alterations induced by JCS1S2 in the treatment of retinal ischemic and hypoxic lesions, thereby offering novel perspectives for further exploration of the pathogenesis and therapeutic targets of related ocular diseases.

## 5 Conclusion

JCS1S2 has great potential as a drug for treating human retinal neovascularization. At present, there are various drugs used in clinical practice to treat neovascularization, but they are expensive, require multiple treatments, and produce heterogeneous treatment responses among patients. Our experiments revealed the following findings: (1) compared with the control group without JCS1S2 injection, intravitreal injection of JCS1S2 can downregulate the expression of VEGF-A, VEGF-B/VEGF-R1, VEGF-A, and VEGF-D/VEGF-R2 mRNA and protein in the retina of OIR rats, suggesting that JCS1S2 can effectively inhibit the proliferation of neovascularization. (2) JCS1S2 may significantly reduce abnormal retinal angiogenesis and Müller cell activation in OIR rats through the TLR4/p-NF-κB/VEGF pathway, and JCS1S2 may have therapeutic potential for retinal neovascularization. The results provide a scientific basis for elucidating the anti-VEGF mechanism of JCS1S2 and developing new treatment strategies for angiogenesis-related diseases. In the future, the pathogenesis and therapeutic targets of JCS1S2 in the treatment of retinal ischemic and hypoxic lesions will be further explored.

## Data Availability

The data presented in the study are deposited in the NCBI repository, accession number PRJNA1243872.

## Abbreviations

AMD, age-related macular degeneration; DEGs, differentially expressed genes; DR, diabetic retinopathy; GO, Gene Ontology; IL-12, interleukin-12; OIR, oxygen-induced retinopathy; PIGF, placental growth factor; RNA-seq, RNA sequencing; ROP, retinopathy of prematurity; TLR, Toll-like receptor; TLR4/NF-κB, Toll-like receptor 4/nuclear factor-κB; TNF-α, tumor necrosis factor-α; VEGF, vascular endothelial growth factor.
